# Supplementary material for: Microporous polymer adsorptive membranes with high processing capacity for molecular separation
Source: Nat Commun. 2022 Jul 19;13:4169. doi: 10.1038/s41467-022-31575-y (PMC9296620; doi:10.1038/s41467-022-31575-y)
Supplement: Supplementary file 1 — Supplementary Information [file 41467_2022_31575_MOESM1_ESM.pdf]

Microporous Polymer Adsorptive Membranes with High Processing Capacity for Molecular Separation

Zhenggong Wang[1], Xiaofan Luo[2], Zejun Song[2], Kuan Lu[3], Shouwen Zhu[1], Yanshao Yang[2], Yatao Zhang,[4] Wangxi Fang,[2] and Jian Jin[1]**

[1]Innovation Center for Chemical Science, College of Chemistry, Chemical Engineering and Materials Science & Collaborative Innovation Center of Suzhou Nano Science and Technology, Soochow University, Suzhou 215123, China.

[2]i-Lab, Suzhou Institute of Nano-Tech and Nano-Bionics, Chinese Academy of Sciences, Suzhou 215123, China.

[3]State Key Laboratory of Coal Conversion, Institute of Coal Chemistry, Chinese Academy of Sciences, Shanxi 030001, China.

[4]School of Chemical Engineering and Energy, Zhengzhou University, Zhengzhou 450001, China.

E-mail: wxfang2017@sinano.ac.cn (W. Fang); jjin@suda.edu.cn (J. Jin)

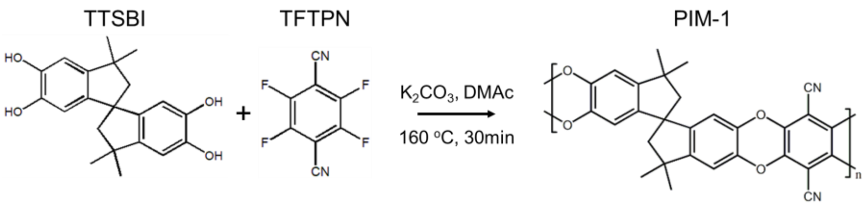

Supplementary Fig. 1 Synthesis route of PIM-1.

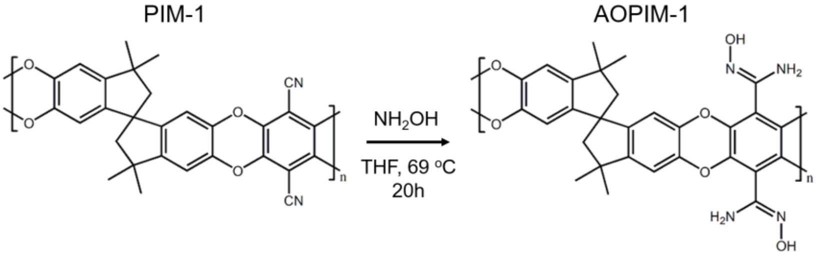

Supplementary Fig. 2 Synthesis route of AOPIM-1.

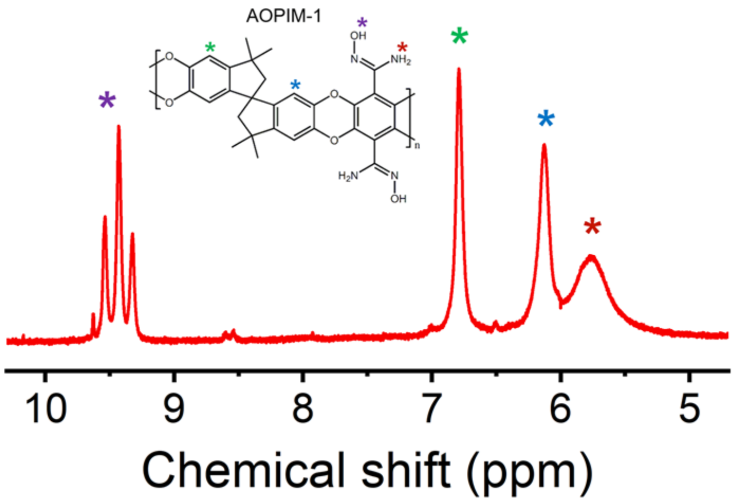

Supplementary Fig. 3 ^{1}H NMR spectrum of AOPIM-1.

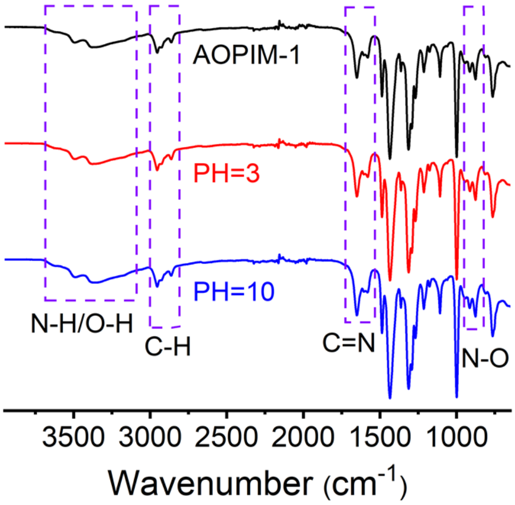

Supplementary Fig. 4 FTIR-ATR spectra of AOPIM-1 as-prepared and after immersing in acid (pH = 3) or base (pH = 10) for at least 24 h.

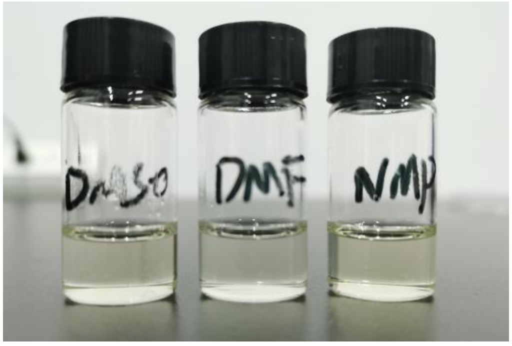

Supplementary Fig. 5 Optical photo of AOPIM-1 solved in different polar solvents.

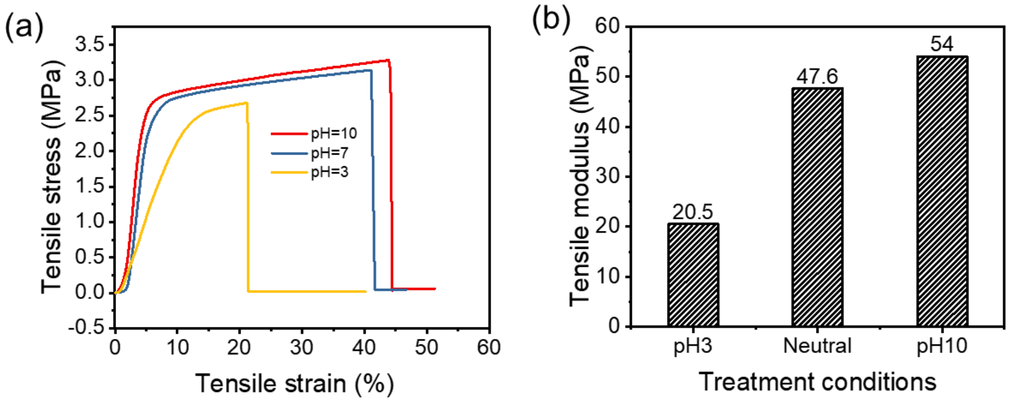

Supplementary Fig. 6 (a) tensile stress curve and (b) tensile modulus of AOPIM-1 membranes (water soaked) under different treatment conditions.

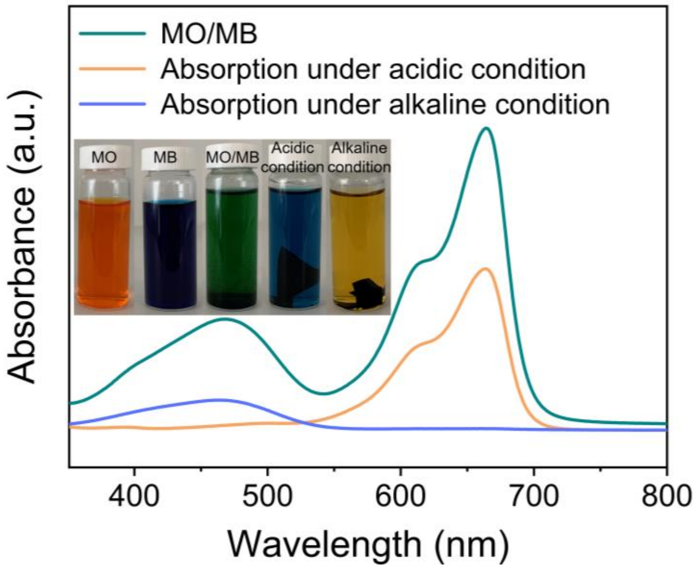

Supplementary Fig. 7 Selective adsorption of AOPIM-1 in MO/MB mixed solutions at different pH conditions and corresponding UV-Vis spectra before and after adsorption.

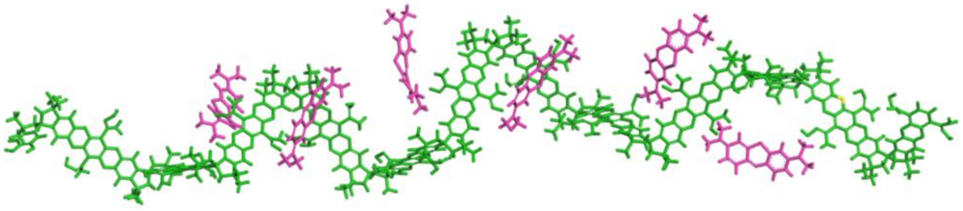

$E_{ads(MB)}$ = -1587.7 kcal/mol

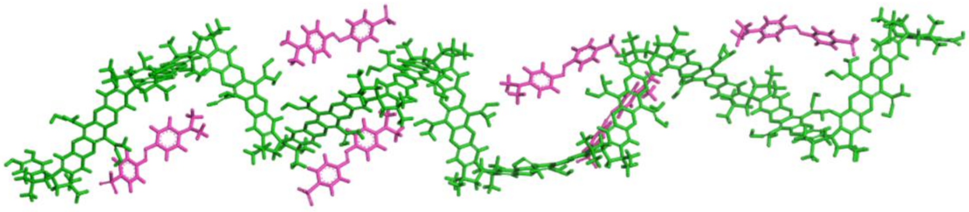

$E_{ads(MO)}$ = -745.4 kcal/mol

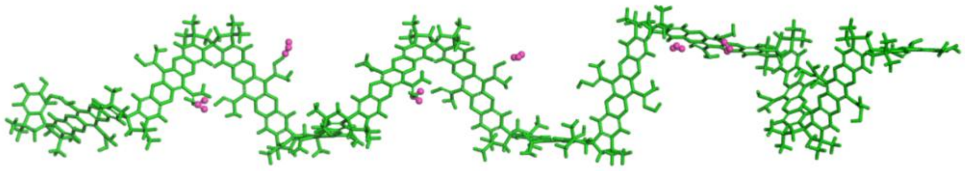

$E_{ads(water)}$ = -742.3 kcal/mol

Supplementary Fig. 8 Energy optimized molecular model and adsorption energies between AOPIM-1 and MB, MO and H_2O molecule.

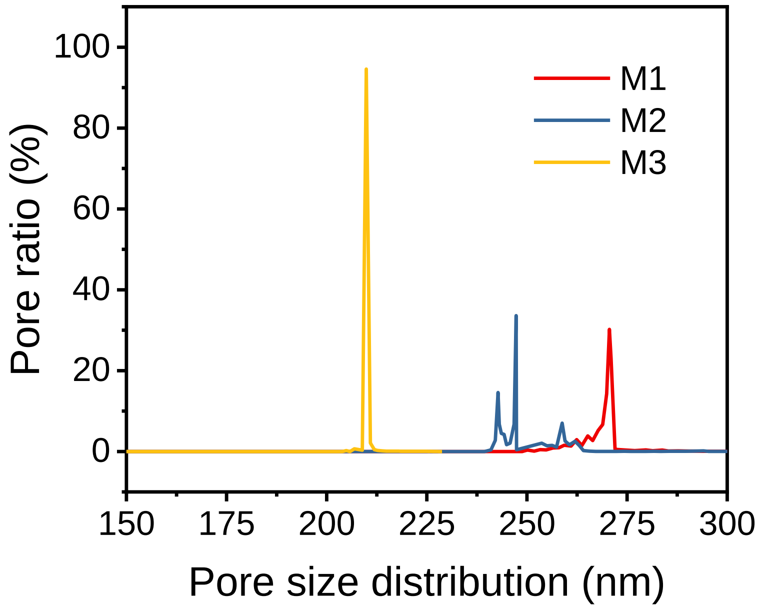

Supplementary Fig. 9 Pore size of membranes prepared in different coagulation baths.

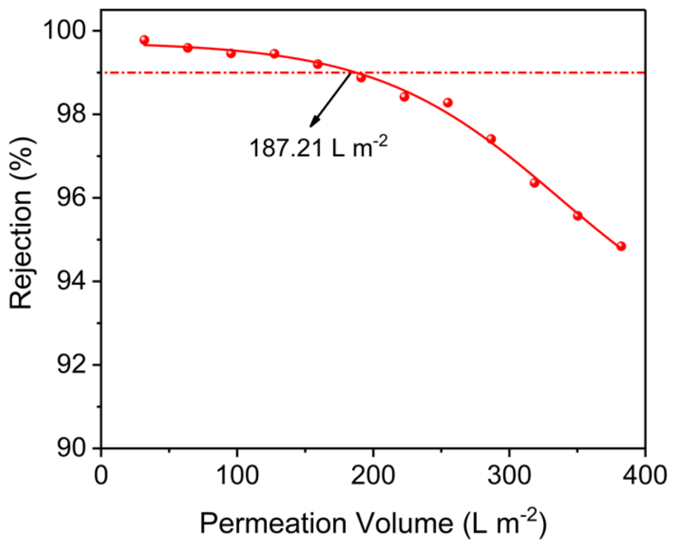

Supplementary Fig. 10 Processing capacity of AOPIM-1 membranes (coagulation bath composition: H_2O : EtOH=100:0).

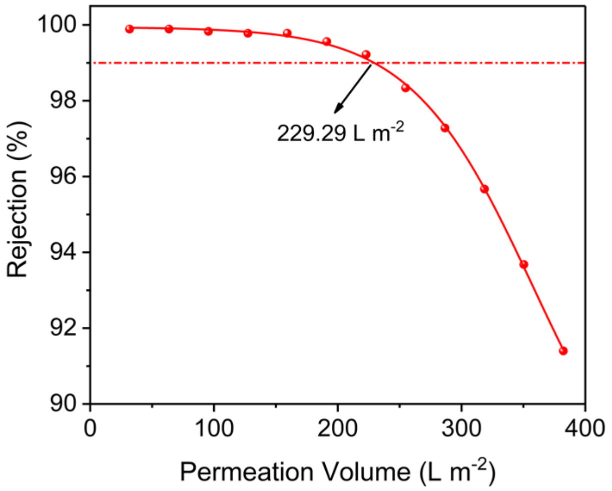

Supplementary Fig. 11 Processing capacity of AOPIM-1 membranes (coagulation bath composition: H_2O : EtOH=50:50).

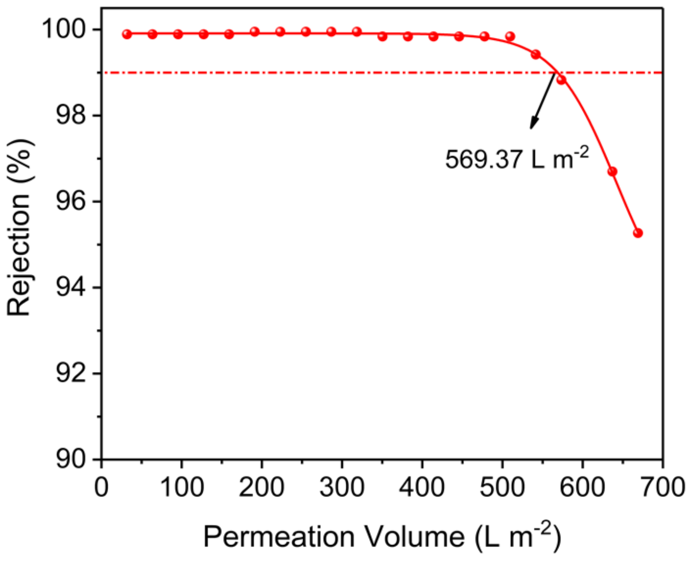

Supplementary Fig. 12 Processing capacity of AOPIM-1 membranes (coagulation bath composition: H_2O : EtOH=0:100).

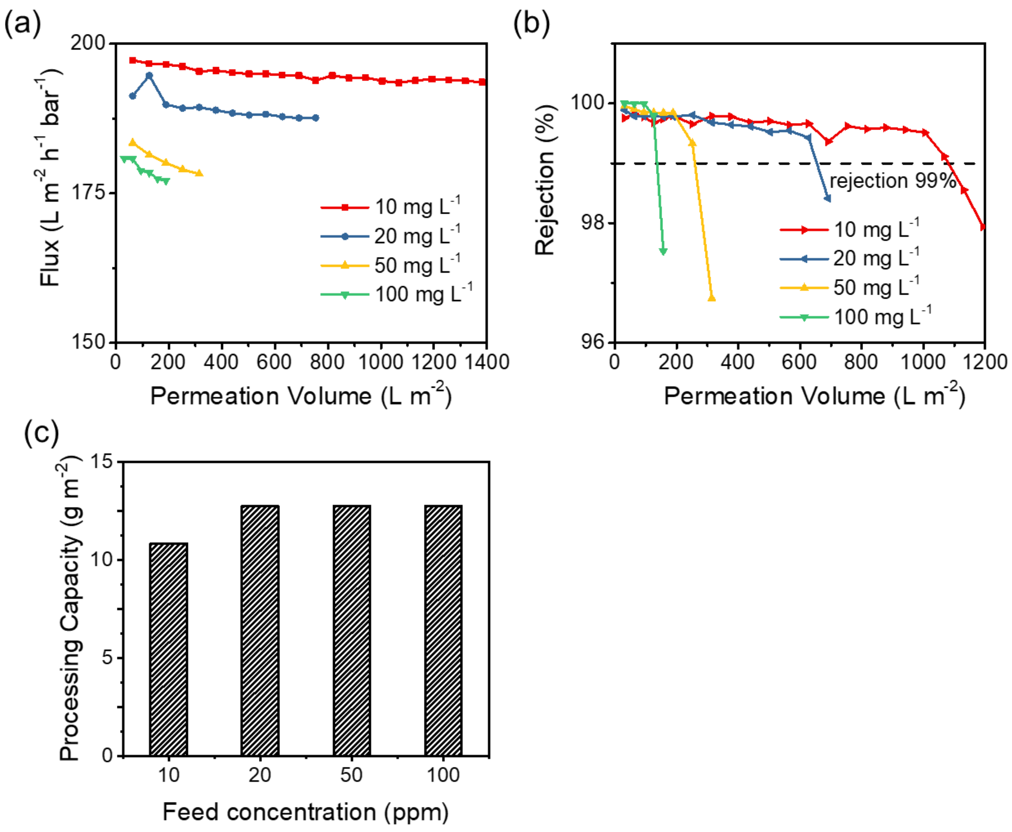

Supplementary Fig. 13 Effects of different concentrations of dyes on membrane flux (a), rejection (b), and adsorption capacity (c).

The solutes concentration influence on the membrane separation performance is further investigated by separating 10/20/30/50 mg L^{-1} RHB solution. The flux of the 10/20/30/50 mg L^{-1} RHB solution varies from 197.21 L m^{-2} h^{-1} bar^{-1} to 193.46 L m^{-2} h^{-1} bar^{-1}, from 191.25 L m^{-2} h^{-1} bar^{-1} to 187.79 L m^{-2} h^{-1} bar^{-1}, from 183.39 L m^{-2} h^{-1} bar^{-1} to 178.97 L m^{-2} h^{-1} bar^{-1} and from 180.78 L m^{-2} h^{-1} bar^{-1} to 178.44 L m^{-2} h^{-1} bar^{-1}. And the permeation volume (rejection > 99%) of 10/20/30/50 mg L^{-1} RHB solution is 1067.6, 628.1, 251.2, 125.6 L m^{-2}, respectively. The mean flux of membrane slightly decreases with increase of RHB solution concentration, while the adsorption capacity is almost unchanged (11.2-12.7 g m^{-2}). Obviously, the concentration of solutes has very little influence on the separation performance of our adsorption membrane. It just has a logical relationship with the adsorption saturation time.

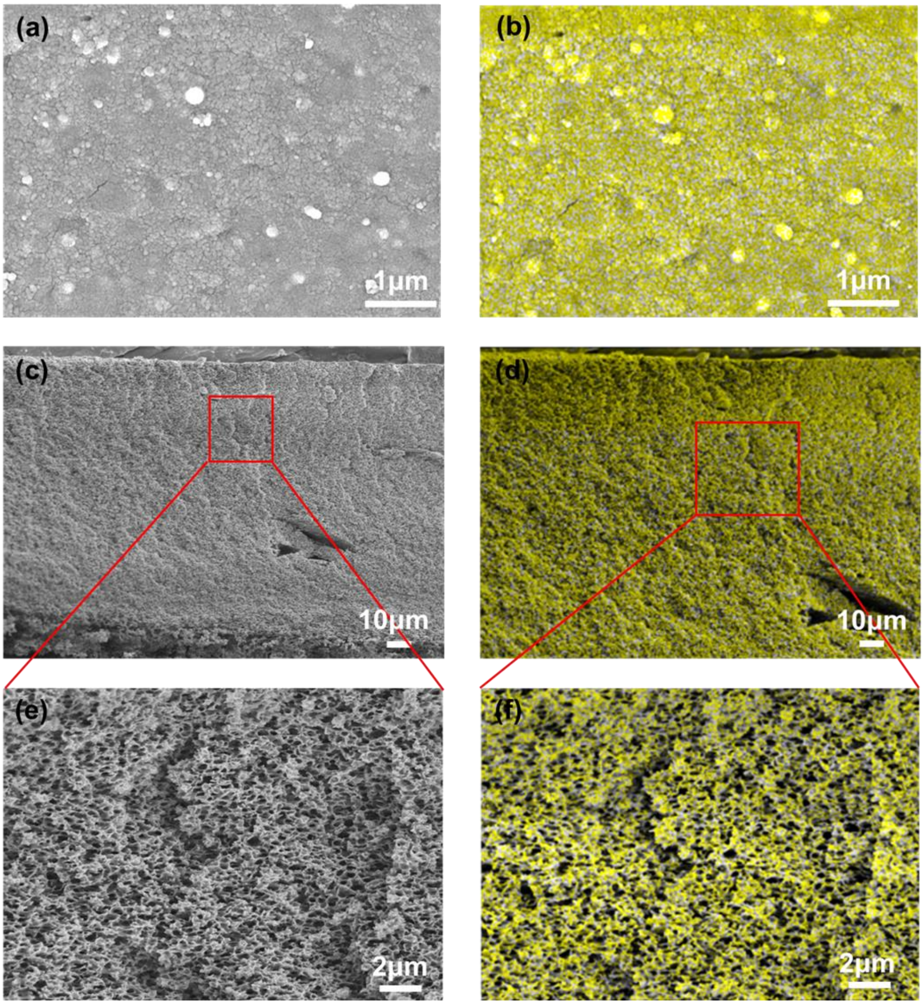

Supplementary Fig. 14 The surface and cross-sectional (a,c,e) SEM images and sulfur element distribution (b,d,f) in EDX mapping images of AOPIM-1 membrane after the dynamic adsorption experiment.

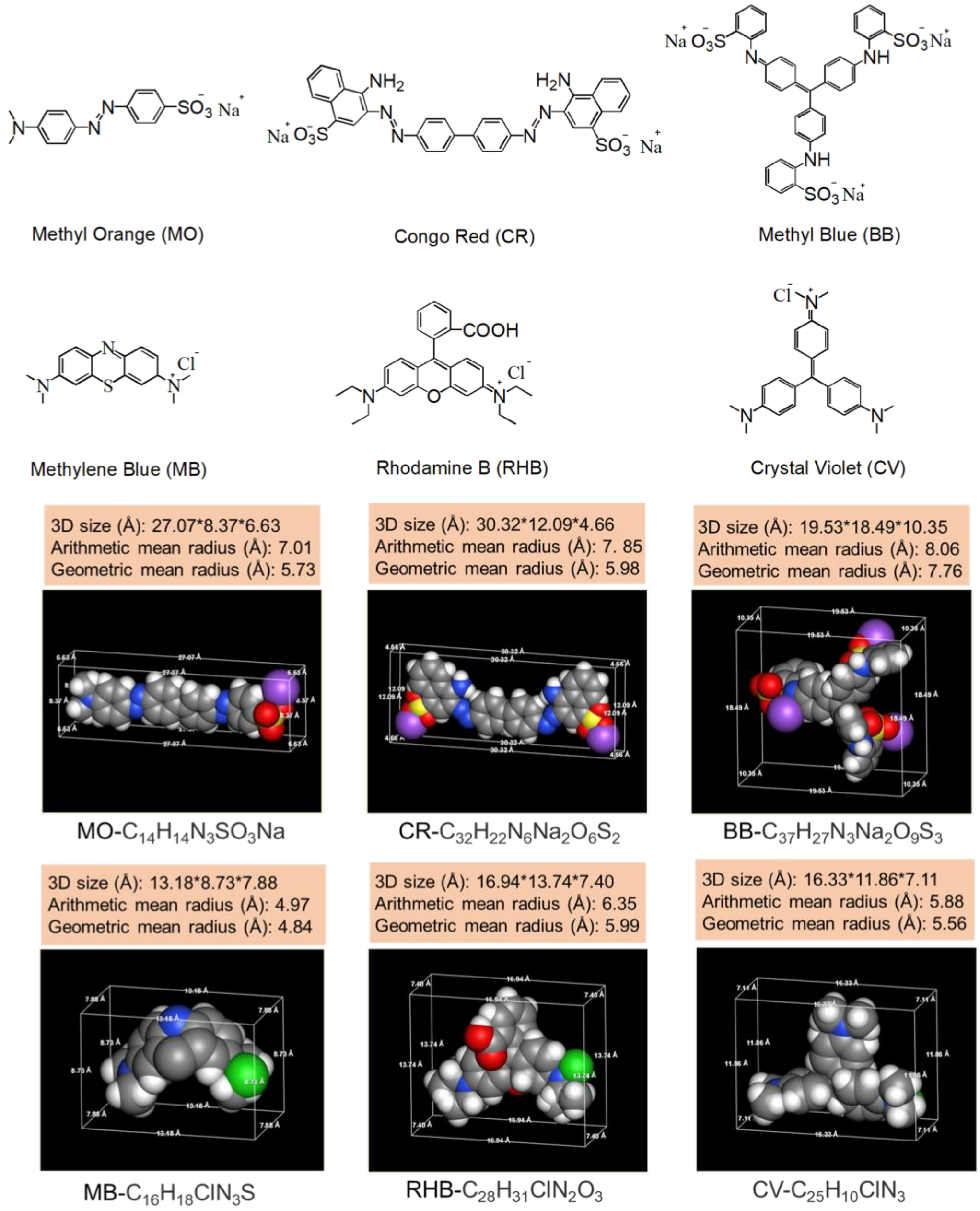

Supplementary Fig. 15 Molecular formula and model size (Å) of different dyes referred in this work.

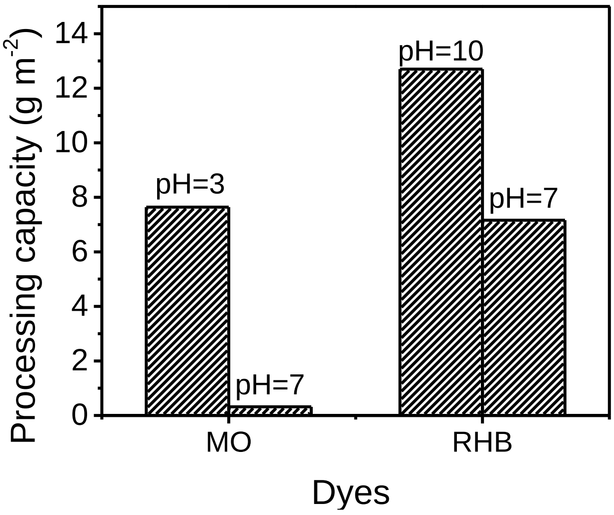

Supplementary Fig. 16 Membrane processing capacity of RHB in in neutral and alkaline condition.

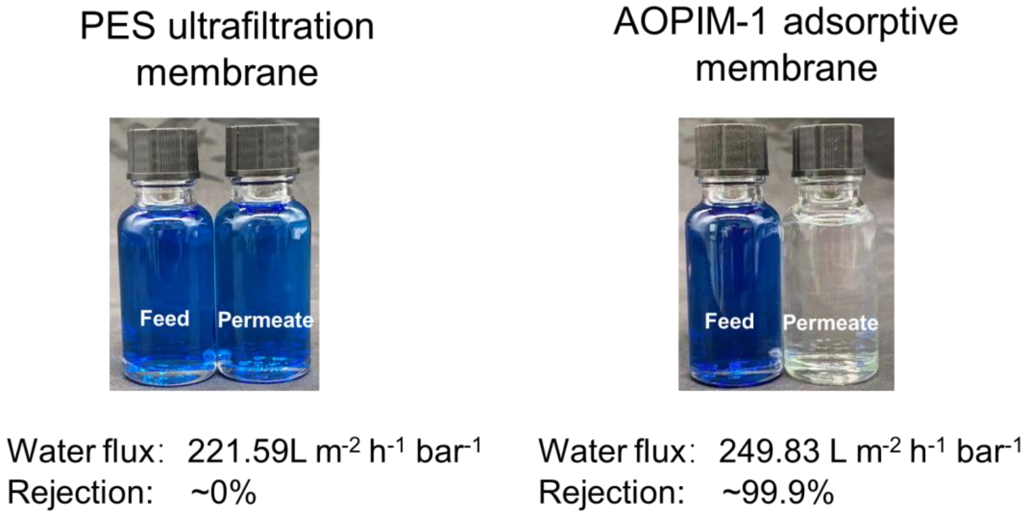

Supplementary Fig. 17 Separation performance of polyethersulfone ultrafiltration membrane and AOPIM-1 membrane.

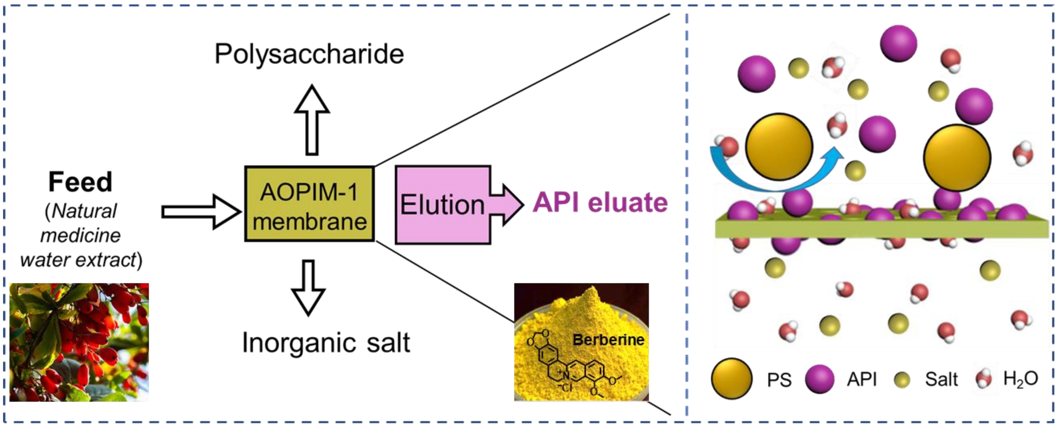

Supplementary Fig. 18 Separation mechanism of natural medicine water extract (APIs/polysaccharides/salt) by AOPIM-1 membranes.

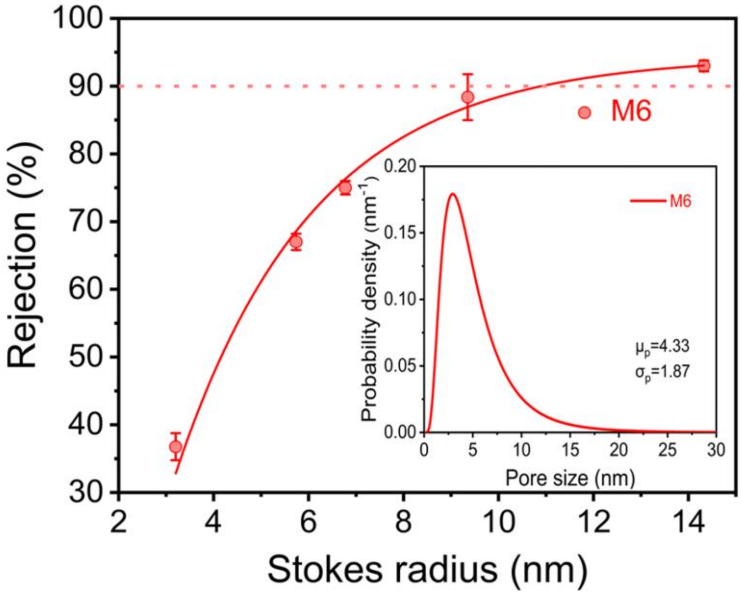

Supplementary Fig. 19 MWCO curve and pore size distribution of M6.

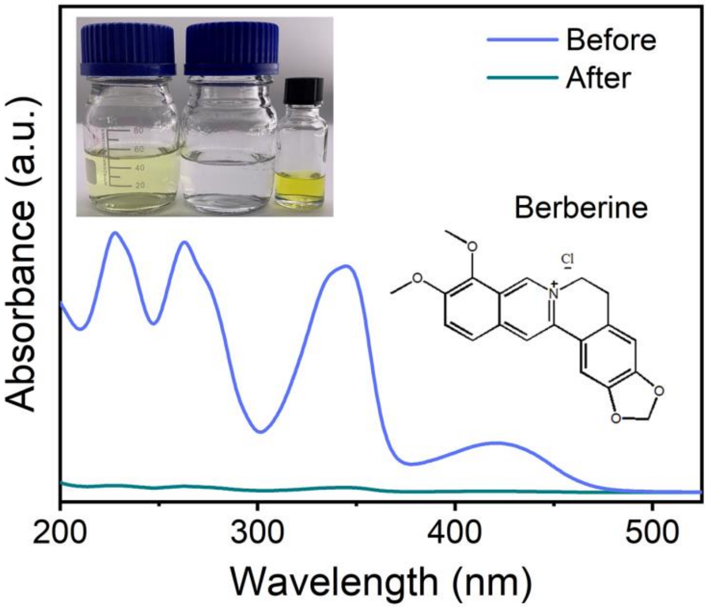

Supplementary Fig. 20 UV-Vis absorption peaks of the stock solution and filtrate before and after the treatment of 50 mL feed. The inset is the optical photos of the stock solution, filtrate, and eluate from left to right, respectively.

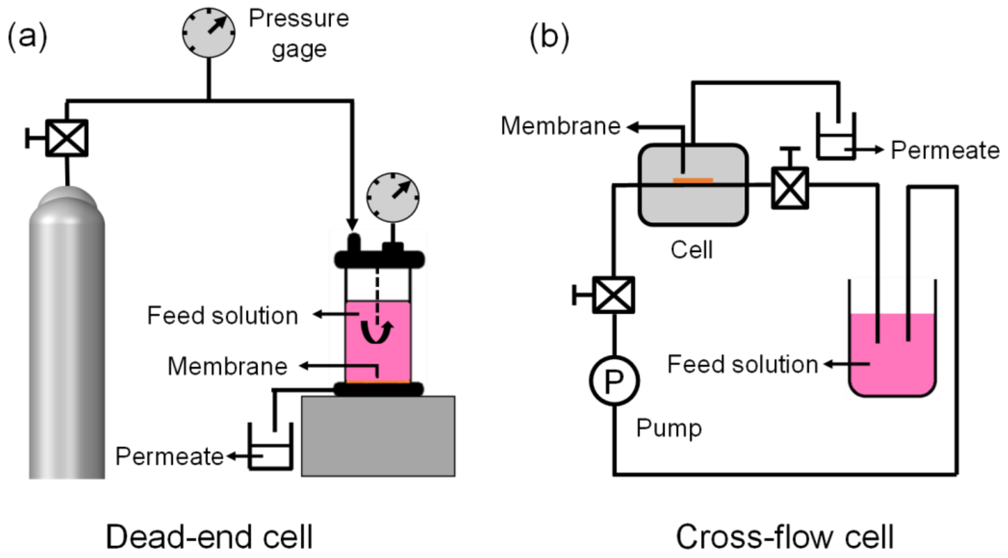

Supplementary Fig. 21 Schematic diagram of Dead-end cell and cross-flow cell used in dynamic membrane adsorption.

Supplementary Table 1 Comparison of adsorption capacity of various adsorbents reported in the literature.

Adsorbents	Chargeability	Specific surface area (m^2 g^{-1})	Adsorption capacity (mg g^{-1})	Adsorbate	Ref.
NH_2-UIO-66	Positive	1035	697.7	CR	20
MIL-100 (Cr)	Negative	3100	507.7	CV	20
ZIF-8@CS sponge	Negative	-	987.0	CR	42
Hydrolyzed PIM-1	Positive	-	424.8	MB	34
PIM-1	Neutral	786	42.3<br>~4<br>~1	MO<br>MB<br>MO	34
Ethanolaminemodified PIM-1	Negative	--	525	Acid Red I	43
AOPAN nanofibrous	pH-response	--	~72	MO	44
AOPIM-1	Netural	--	86.7<br>81.3	MO<br>MB	40
PES nanofibrous membrane	Positive	20	208	CR	22
PES/MS	Netural	--	602.3	MB	46
PQAM nanofibrous membrane	Positive	--	909.8	MO	45
AOPIM-1	pH-response	550	491.63	MO	This work
			765.09	MB	

Supplementary Table 2 Properties of asymmetric membranes under different preparation conditions.

Membrane	Casting parameters		Thickness (μm)	Pure water permeance (L m^{-2} h^{-1} bar^{-1})	Processing capacity (g m^{-2})	Structure type
	Concentration	Coagulation bath (H_2O:EtOH)				
M1	13 wt%	100:0	131 ± 5	1505.73 ± 326.33	3.7	Finger-like
M2	13 wt%	50:50	102 ± 2	733.26 ± 146.89	4.6	Finger-like
M3	13 wt%	0:100	64 ± 4	249.83 ± 34.01	11.4	Sponge-like
M4	15 wt%	0:100	93 ± 2	211.81 ± 18.31	17.8	Sponge-like
M5	18 wt%	0:100	119 ± 6	183.30 ± 8.43	26.1	Sponge-like

Supplementary Table 3 Comparison of processing capacity of various membrane adsorbents reported in the literature.

Membrane adsorption	Adsorbate	Concentration	Pure water Permeance (L m^{-2} h^{-1} bar^{-1})	Processing capacity (g m^{-2})	Reference
ZIF-8 hybrid aerogels	Dyes	3 ppm	480-650	0.48-1.0*	25
β-CDP-40%	BPA	10 ppm	400	1.150*	26
β-CDP composite porous membrane	BPA	10 ppm	1300	0.440	27
CNF-100	MB	20PPM	5300	2	28
PES	MB	20PPM	221.6	~0	This work
AOPIM-1	RHB	20 ppm	249.8	11.4	This work
			211.8	17.8	This work
			183.3	26.1	This work

Supplementary Table 4 Summary of the dynamic adsorption performance of AOPIM-1 membrane.

pH condition	Dyes	Concentration (ppm)	Molecular weight (Da)	Permeance (L m^{-2} h^{-1} bar^{-1})	Rejection (%)
	MO	20	327.3	203.82 ± 2.46	99.9
pH = 3	CR	20	696.6	180.92 ± 4.17	99.9
	BB	20	799.8	192.50 ± 0.89	99.9
	MB	20	319.8	177.9 ± 2.11	99.9
pH = 10	RHB	20	479.0	191.31 ± 1.17	99.9
	CV	20	407.9	170.00 ± 1.85	99.9

Supplementary Table 5 Concentration of the feed, permeate and eluent (about 20 ml) in each cycle.

Cycle	1	2	3	4	5	6	7	8
**Feed (ppm)**	20	20	20	20	20	20	20	20
**Permeate (ppm)**	0.56	0.63	0.52	0.77	0.85	1.18	1.40	0.99
**Eluent (ppm)**	153.6	151.7	151.5	149.4	150.6	145.2	157.7	--

Supplementary Table 6 Composition of synthetic water extract feed solution.

Materials	Concentration (ppm)
NaCl	1000
Polysaccharide	200
Berberine	20
